# “Get to Know My Community”: Community‐Driven Guidance for Future Transgender and Nonbinary Focused Research

**DOI:** 10.1002/jcop.70118

**Published:** 2026-06-30

**Authors:** Kye Campbell‐Fox, Jae A. Puckett, Arden Henderson, Devon Kimball, Lauren Wiklund

**Affiliations:** ^1^ Department of Psychology Michigan State University East Lansing MI USA

**Keywords:** community‐engaged, minority stress, nonbinary, resilience, transgender

## Abstract

Research pertaining to transgender and nonbinary (TNB) populations has not always reflected the concerns of TNB people and some research has perpetuated harm. Research should be guided by TNB communities to identify areas of key importance to these populations. We asked TNB focus group participants to share their perspectives on important areas for future research with TNB populations. We coded responses across groups using reflexive thematic analysis. Four themes were developed: (1) Enhance the Quality and Inclusivity of TNB Research, (2) Understand the Diversity of TNB Communities, (3) Document and Support TNB Survival in a Cisnormative System, and (4) Explore TNB Thriving. These findings highlight important topics that future research can prioritize. This work adds to a growing body of literature that emphasizes the benefits of community‐engaged research and can guide future studies to better address TNB people's needs.

## 1 Introduction

Some research has perpetuated pathologizing and stigmatizing views of transgender and nonbinary (TNB) communities, resulting in a complicated history that continues to shape knowledge and some community members' perspectives of research (Adams et al. 2017; Edmiston 2018; Rajkovic et al. 2022; Vincent 2018). Some research has even caused harm to TNB people and communities through the pathologization of TNB identities. For example, some research built on outdated and cisnormative understandings of gender has been used to deny TNB people access to gender affirming medical care, to promote conversion therapy practices, and to invalidate TNB people's identities (Adams et al. 2017; Edmiston 2018; Rajkovic et al. 2022; Vincent 2018). Most research has shifted away from treating TNB identities as deviations, but many current practices still fail to reflect the research goals and needs of TNB communities (LeBlanc et al. 2022; Marshall et al. 2022). Research can benefit from involving TNB people in determining research topics that are of importance to these communities. Community‐engaged and participatory research methods offer a way for TNB communities to be more involved in guiding research and setting research priorities that will address the most pressing issues facing TNB communities (Fahlberg 2023; Hughes and Martino 2023; Katz‐Wise et al. 2019; Rodriguez Espinosa and Verney 2021; Singh et al. 2013; Travers et al. 2013). In service of this vision, we report on data from focus groups regarding TNB people's feedback for future TNB‐focused research.

There are many reasons to move from doing research *on* TNB people to *with* TNB people (Rodriguez Espinosa and Verney 2021; Rosenberg and Tilley 2021; Veale et al. 2022). Gathering community input throughout every stage of the research process provides insight that creates an ethical and inclusive study from its inception (Fine et al. 2021; Katz‐Wise et al. 2019; Rosenberg and Tilley 2021; Veale et al. 2022). A direct way to ensure research is representative of TNB community priorities is to have community members and researchers collaborate as co‐leaders, or to have entirely TNB‐led projects (Rosenberg and Tilley 2021; Veale et al. 2022). Integrating community members into the research process can disrupt power imbalances inherent to research by empowering community members to make decisions about the aims and scope of studies done in their communities (Hope et al. 2022; Katz‐Wise et al. 2019; Singh et al. 2013; Travers et al. 2013). Furthermore, community‐engaged and participatory research tends to integrate a social justice approach in which research seeks to make a tangible impact on the lived experiences of participants or communities to address inequities rather than simply generating knowledge for academic gains (Fine et al. 2021; Four Corners TNB Health Research Advisory Network 2021; Rodriguez Espinosa and Verney 2021). Considering these benefits, more research is needed that integrates the perspectives of TNB community members.

As mentioned, one way to facilitate this involvement of community members is to inquire about or co‐develop research aims that are viewed as important to TNB communities. Others have shown that there are areas viewed as understudied by TNB communities. In one area, health, there are a variety of topics that have been understudied, such as experiences in healthcare (Veale et al. 2022) and holistic healthcare models that consider one's broader healthcare needs within the context of TNB‐specific care (Four Corners TNB Health Research Advisory Network 2021; Morse et al. 2023). Another understudied topic is reproductive and sexual health, particularly TNB fertility preservation and family planning (Four Corners TNB Health Research Advisory Network 2021; Misiolek et al. 2020; Veale et al. 2022; Wanta and Unger 2017). In addition, barriers to healthcare, like a lack of health insurance or affordable care options (Four Corners TNB Health Research Advisory Network 2021; Edmiston et al. 2016; Misiolek et al. 2020; Restar et al. 2023), are an understudied factor in TNB health research. There is also a strong need for research on the drivers of health disparities, including the influence of minority stressors in relation to TNB people's health and well‐being (LeBlanc et al. 2022; Marshall et al. 2022; Restar et al. 2023; Veale et al. 2022). Bolstering research on these topics could pave the way to improved access to care, more affirming approaches to healthcare services, or reductions in health disparities. More community involvement is warranted in shaping how these topics are explored or what specific research questions would be the most impactful from the perspectives of community members.

TNB people have also called for research to be reflective of their full lived experiences, including positive topics. For example, there have been calls for greater research on resilience, empowerment, advocacy, community strength and connection, and affirmation (Four Corners TNB Health Research Advisory Network 2021; Hughes and Martino 2023; Katz‐Wise et al. 2019; LeBlanc et al. 2022; Misiolek et al. 2020; Rodriguez Espinosa and Verney 2021; Singh et al. 2013; Thompson et al. 2024; Veale et al. 2022). When research focuses narrowly on TNB people's negative experiences, it can paint a one‐sided picture of their lives, inherently reinforcing stereotypes or creating narratives that perpetuate internalized stigma (Levitt et al. 2023). Research about TNB people's strength and resilience has the possibility to offset these limited understandings about TNB communities.

Recent reviews also highlight the underrepresentation of positively focused TNB research. Reviews have shown that there has been substantial growth in research overall about TNB communities. A bibliometric analysis spanning 1994–2026 found that there were notable increases after 2016, with the most TNB‐focused research published in 2024 (Anjaly and Thanuskodi 2026). Their review also found that there have been shifts in the research topic trends over time, with work shifting from exploring TNB identity to a focus on gender affirming care and, in more recent years, more expansive research on topics like equity and legal rights. In multiple bibliometric reviews, analyses have shown that HIV, mental health, discrimination, stigma, therapy or counseling, and gender dysphoria have been prominent topics within the field (Anjaly and Thanuskodi 2026; Arnull et al. 2023; Sweileh 2018, 2022). Within the topic of mental health, there has been an increase in the focus on resilience (Anjaly and Thanuskodi 2026), showing some growth in more positively framed research, potentially. Even so, Sweileh (2022) found that resilience research only represented 2.5% of the mental health‐focused research from 1992 to 2021. Although there has been growth in these areas, there is still much room for improvement (Levitt et al. 2023) and shifts are needed to reflect a fuller understanding of TNB people's lived experiences.

Another way in which TNB research can better reflect the lives of TNB people is by more fully attending to the diversity of experiences represented in these communities. This can partially be addressed by incorporating intersectionality into more TNB‐focused research (Wesp et al. 2019). Intersectionality calls for greater consideration of how systems of power (i.e., privilege and oppression) influence lived experiences and how these experiences are shaped by various aspects of identity – not just gender (Crenshaw 1991). Intersectional perspectives can work to contextualize TNB people's experiences and move away from viewing differences as solely individual experiences or personal challenges devoid of connections to systemic factors and instead move researchers to recognize the role of structural factors and power in shaping one's experiences (Adams et al. 2017; Four Corners TNB Health Research Advisory Network 2021; LeBlanc et al. 2022; Misiolek et al. 2020; Morse et al. 2023; Olson‐Kennedy et al. 2016; Owen‐Smith et al. 2016; Restar et al. 2023; Safer 2021; Singh et al. 2013; Thompson et al. 2024; Vincent 2018). There is also diversity within the TNB community to consider, such as different experiences between nonbinary people and trans men and women, as well as the experiences of gender groups specific to Indigenous communities (Adams et al. 2017; Four Corners TNB Health Research Advisory Network 2021; Edmiston et al. 2016; Fahlberg 2023; Marshall et al. 2022).

### 1.1 Current Study

Given the lack of TNB inclusion in the planning and implementation of most research studies (Rosenberg and Tilley 2021; Streed et al. 2023), it is critical to seek community guidance on areas of importance. This helps to ensure that research is focused on topics that TNB communities will find important and useful in their lives. Furthermore, these priorities can change over time given shifts that happen in the social and legal landscape of TNB people's lives, making it important to continuously pursue input from TNB communities on research topics and the most affirming practices. In the current study, we gathered data on TNB people's feedback on future research directions via focus groups to help further guide affirming TNB research studies.

## 2 Methods

The current study used focus groups to gather input on the minority stress experiences of TNB people and feedback on future research directions, with the latter the focus of this manuscript. This represents a secondary data analysis of the information gathered in the broader study. Participants were recruited via online advertisements, social media websites (e.g., Facebook, Instagram), and outreach to community organizations (e.g., LGBTQ+ community centers) to distribute the study flyer (both virtually and in‐person), such as through their email listservs or social media. We took a variety of steps to ensure the quality of our data due to the prevalence of issues with bots and fraudulent responders online, such as requiring a screener questionnaire prior to enrollment in the study, reviewing responses for duplicate entries, reviewing IP addresses for suspicious locations, reviewing attention check questions, and reviewing response bursts carefully. We used purposive sampling to ensure representation across a range of genders, ages, and racial and ethnic identities. The final sample included 72 participants, who reported a range of TNB gender identities (see Table 1 for demographic information).

**Table 1:** Sample Demographics.

Characteristic	Full Sample *N* (%)
Gender Identity	
Non‐binary	25 (34.7%)
Trans Woman	20 (27.8%)
Trans Man	14 (19.4%)
Genderqueer	4 (5.6%)
Genderfluid	3 (4.2%)
Agender	2 (2.8%)
Woman	1 (1.4%)
Man	1 (1.4%)
Not listed	2 (2.8%)
Sex Assigned at Birth	
Female	45 (62.5%)
Male	27 (37.5%)
Difference of Sex Development (also known as intersex)	
No	57 (79.2%)
Yes	1 (1.4%)
Unsure	14 (19.4%)
Race or Ethnicity	
White	33 (45.8%)
Multiracial/Multiethnic	23 (31.9%)
Black or African American	13 (18.1%)
Latino/a/x	2 (2.8%)
Asian	1 (1.4%)
Native American, American Indian, or Alaskan Native	0
Native Hawaiian or Other Pacific Islander	0
Sexual Orientation	
Queer	24 (33.3%)
Bisexual	18 (25.0%)
Pansexual	11 (15.3%)
Lesbian	5 (6.9%)
Asexual	5 (6.9%)
Heterosexual/Straight	4 (5.6%)
Gay	2 (2.8%)
Not Listed	3 (4.2%)
Income	
Less than $10,000	22 (30.6%)
10,000–19,999	11 (15.3%)
20,000–29,999	13 (18.1%)
30,000–39,999	5 (6.9%)
40,000–49,999	7 (9.7%)
50,000–69,999	4 (5.6%)
70,000–99,999	5 (6.9%)
100,000 or more	5 (6.9%)
Disability	
Yes	36 (50%)
No	29 (40.3%)
Prefer Not to Say	7 (9.7%)
Geographical Area of Residence	
More Urban	38 (52.8%)
More Rural	8 (11.1%)
More Suburban	26 (36.1%)

After reviewing the screener survey, participants were invited to the focus groups. The focus groups each lasted approximately 2.5 h and were modeled after the participatory focus group approach, Youth GO (Generate, Organize, hereafter called GO, given our use with adults; Stacy et al. 2018; Stacy et al. 2020). This is a multi‐step approach to generating data and engaging participants in the analysis of this data. The study was approved by the Michigan State University Institutional Review Board.

First, participants were sent informed consent documents prior to the focus groups, along with a brief demographics questionnaire (age was asked as a text box, all other items were presented with the response options shown in Table 1). At the time of the interview, we reviewed the informed consent information, followed by creating a set of shared community agreements (e.g., giving space for others to talk). We then used an icebreaker to build rapport and engagement with participants. The bulk of the focus groups were centered on discussing experiences of minority stress, with a subsection focused on recommendations for future research more generally. We used Google Jamboards, a virtual collaborative tool entailing a whiteboard and sticky note features, to present each prompt.

The prompt that we focus on in the present manuscript was: “Is there anything you want to share to help inform future research about trans and nonbinary people's experiences?” Participants were given time to reflect on their answers and write down responses using the sticky note function on the Jamboards. After that, we engaged in a group discussion of the prompt and responses, providing space for participants to elaborate and discuss their perspectives with each other. Participants were paid $75 via electronic gift cards.

### 2.1 Analysis

We used reflexive thematic analysis to generate themes about participants' feedback on future research directions, with slight adaptations to facilitate a team coding approach (Braun and Clarke 2006; Braun and Clarke 2022). The coding team consisted of the first, second, and third authors. First, we familiarized ourselves with the data through repeated readings of the transcripts, as well as a review of the video recordings and images of the Jamboards. We then created a code list through an iterative and reflexive process. This entailed reviewing the data multiple times, writing memos, and engaging in group discussions about common ideas repeated in the data. This ongoing and evolving code list was then applied to the data with additional review and revision of the coding by the team. After all coding was applied, the second author reviewed all code applications to ensure they reflected the intended meanings of the codes. Small adjustments were made as needed to align with the code list. Another review was then conducted, which did not identify any further edits needed to align the coding with the code list. After this, we compared the codes to each other to identify broader themes that reflected clusters of codes with a shared underlying concept or idea. Team members developed preliminary ideas for themes, which we then revised as a group. Our group reflections helped to ensure that our themes adequately described the data and deepened our reflexive analysis. Furthermore, we reviewed all of the codes and the data within each theme to ensure that the themes described the experiences referenced in the data. We also ensured that there were clear boundaries delineated between themes.

We took an inductive approach to the analysis, generating codes and themes that were grounded in the data. Our analysis was driven by an experiential framework as we sought to prioritize participant experiences and voices in our analysis. We approached the analysis with a shared understanding about the importance of TNB communities being given agency over their lives and over the research done about their experiences. As such, we sought to reflect participants' experiences and what they desired for future research. We also recognize that there are many diverse perspectives within these communities, and we sought to understand that diversity rather than assume a singular experience for TNB people. The research team included multiple TNB individuals, as well as cisgender individuals. We reflected throughout the process on how our identities may have influenced the experiences of participants during data collection and our individual interpretations of data during analysis.

## 3 Results

We generated four themes to encompass participants' suggestions: (1) Enhance the Quality and Inclusivity of TNB Research, (2) Understand the Diversity of TNB Communities, (3) Document and Support TNB Survival in a Cisnormative System, and (4) Explore TNB Thriving. Quotes are provided throughout the results to demonstrate the findings. When these quotes were from verbal statements in the focus groups, we provided information about the participants' age, gender, and race/ethnicity. Some quotes were obtained from participants' written responses on the sticky notes and, as such, these cannot be attributed to a specific participant.

### 3.1 Enhance the Quality and Inclusivity of TNB Research

Participants discussed marginalization that can be experienced within the context of research studies, particularly when these studies lack TNB input or leadership. As such, participants noted that research practices need to be improved overall to enhance the quality and inclusivity of research. Participants recommended that cisgender researchers who work with TNB communities need to educate themselves about TNB identities beforehand, including recognizing the long history of TNB identities across many cultures and the diversity of experiences within TNB communities. Education is especially important regarding inclusive language for asking about gender, sex assigned at birth, and gender modality. For example, one participant wrote that they “feel uncomfortable” with selecting the term “other” rather than a more inclusive term, while another participant wrote that “gender options need to be significant [in number],” and should always include the option to self‐describe. Participants felt that researchers should also ensure they recognize the range in gender identities that exist, including nonbinary identities.

Participants shared that another practical step to overhauling research practices is to have TNB voices represented as leaders and experts on research teams and to use research measures created by and for TNB people. One participant emphasized this by writing, “Nothing about us, without us. Trans people should be at the head of research for us.” Another method mentioned by participants for increasing TNB participation and community benefit is to offer financial incentives for participants' time and effort. Participants noted the importance of offering increased incentives for longitudinal studies – an area sorely lacking in TNB research, but which requires a significant amount of time and effort from participants. Participants reflected that longitudinal studies would enhance the quality of research and incentives would also help them to feel valued as participants.

### 3.2 Understand the Diversity of TNB Communities

“We are not all the same,” one participant wrote, a common refrain across focus groups. Participants expressed frustration with studies that only looked at a small portion of the TNB community and made assumptions that those findings apply broadly to all TNB people. For example, participants mentioned research that heavily focuses on White, financially stable, and transgender people who have undergone medical gender affirmation. Participants also emphasized that TNB communities include people of all backgrounds, whose many identities intersect and intertwine. One participant (32 years old; genderqueer; Latinx and White) shared “I have…Indigenous roots in Chile, where gender identities were more expansive and that was taken away during colonization.” Their gender and ethnicity were inherently interconnected with each other and with the experience of colonization, shaping how their identities were collectively experienced and embodied. Participants drew connections to other understudied aspects of identity, including spirituality, neurodivergence, ability, sex, race, class, age, and culture more generally, calling for more complex and multifaceted research on their lives.

Participants noted that TNB communities are also diverse in terms of gender identity, transition access, transition decisions, and experiences of gender. As one individual (19 years old; genderfluid; White) said, “it's really important that we talk about… how big the trans community really is.” As an example, they explained, “there's also a whole community of trans people who exist and are okay with just not passing.” In addition, participants shared that TNB experiences with medical transition are not monolithic. Participants noted that not all TNB people have access to gender affirming care, that some people choose not to affirm their gender through medical means, and that others may retransition at later points. Participants suggested researching the diverse pathways of affirmation for TNB people that can account for these varied experiences.

### 3.3 Document and Support TNB Survival in a Cisnormative System

Some participants discussed future research areas that pertained to minority stress. Some participants emphasized specific stressors that need more research, such as vicarious stress, workplace stressors, gender dysphoria, and the effects of sociopolitical environments. One participant wrote, “How can I survive, much less build a life, if my state wants to erase me?” Participants also reflected on how just surviving in a cisnormative environment leads to “sheer pervasive exhaustion” (24 years old; non‐binary; White). Participants emphasized that research should capture the complex and interrelated dynamics of TNB people's lives, including the hardships and contexts in which these occur.

Experiences within families came up as a particularly nuanced area to consider as well. Participants indicated that family relationships are more complicated than a dichotomy of accepting or unaccepting. For example, one person's wife was supportive of them, but her family disowned her for marrying a TNB person, which complicated things for them both. Other individuals spoke of TNB parents being denied access to their children or of having limited reproductive and parenthood options in the first place. Participants recommended that future research explore family in more complex and meaningful ways.

Participants also shared that meeting basic needs such as housing, food, and healthcare can be especially difficult for marginalized populations. Participants suggested that research could contribute not only knowledge on these issues, but also practical solutions and tangible support. Participants called on researchers to push beyond documenting problems to providing information that can make a practical difference in the lives of TNB people, such as improving access to TNB‐affirming healthcare services.

### 3.4 Explore TNB Thriving

Many TNB people thrive and live happy and fulfilling lives; however, participants felt that researchers rarely study these experiences. Instead, participants reflected that much of the emphasis in TNB research thus far has been on the deficits and challenges faced by TNB communities. As one participant wrote, “LGBTQ research tends to focus on the negatives, would love to see more research on unique strengths and skills developed by trans people from their experiences that can help/empower others.” One participant (26 years old; genderfluid; White) discussed how limited depictions of TNB people, including in research, mask the possibilities of TNB people's lives. For this participant, their mother's hearing research about the hardships of TNB people contributed to her limited understanding. Reflecting on how this participant felt in response to their mother's perspective, they shared:

Our words create realities… It's so sad to me that you [mom] don't see the expansiveness that is possible when we are outside of…these boxes. And it's sad to me that you [mom] think that is a place of sadness, because it's only a place of sadness because you [mom] are enforcing the boxes.

This individual had experienced harm because of how one‐dimensional presentations of TNB experiences from research can affect cisgender people's views of TNB communities, but they also saw how living outside of gender boxes and embracing expansiveness could lead to a more fulfilling life.

Other participants expressed similar feelings about research and that it often neglects the joy and beauty of TNB identities. Another participant exemplified this, writing “trans people exist outside of suffering.” This comment also encapsulated the frustration with promoting a single TNB narrative that only emphasized pain and marginalization. Participants had specific suggestions for more positive topics, such as community building, resilience, and support. One person suggested in writing the research question “how do trans people build community [and] access social safety networks?” Another wrote that researchers should “Focus on aspects of community among trans people and how it connects to resiliency.” Understanding the factors that drive TNB thriving can expand the diversity of TNB narratives in research.

## 4 Discussion

For research to truly benefit TNB communities, we must consider the unique needs, strengths, and priorities within these communities rather than imposing outsider perspectives or researcher needs onto TNB participants. The history of marginalizing research practices with TNB communities makes this even more critical (Adams et al. 2017; Edmiston 2018; Rajkovic et al. 2022; Vincent 2018). Prioritizing community voice in shaping research priorities and objectives can help to ensure research is of greater value to TNB people and that the most critical areas of concern to the community are being addressed. Below, we provide suggestions for how to integrate the findings from this study into future TNB research.

### 4.1 Centering TNB People

At the core of our findings is the importance of centering TNB people in the research process, aligning with calls from others for TNB people to have meaningful roles in shaping research (Adams et al. 2017; Misiolek et al. 2020; Veale et al. 2022; Vincent 2018). Participants' recommendations highlight the ways that cisgender people's assumptions or priorities may not align with TNB people's lived experiences or needs. Community‐engaged and participatory research practices provide an avenue to address this concern. Community‐engaged research exists along a continuum and could include, on one end, researchers gathering input from TNB community members on an established research plan and, on the other end, research with TNB community members in control of the entire process (Travers et al. 2013). Throughout the process, researchers should carefully consider how they share power with TNB communities (Travers et al. 2013). Researchers may also explore particular methods, such as participatory action research (for a discussion of using participatory action research with TNB communities, see Singh et al. 2013). In addition, many studies are led by cisgender research teams, which calls for critical reflection of how to advance the work of TNB researchers (Riggs et al. 2024; Veale et al. 2022).

We also believe it is critical for cisgender researchers to engage in reflection about their research practices and the impacts that they have on TNB people. Not all biases or lack of knowledge can be mitigated by somehow integrating TNB people into the research process. In truth, it may set TNB people up to experience microaggressions or invalidation to be integrated into research teams that have not engaged in critical reflection in these areas. As such, it is essential that cisgender researchers put in the effort to educate themselves about TNB people's lived experiences without burdening TNB people to be their educators, that they reflect on the privileges they hold as cisgender people, and that they take genuine steps towards allyship to TNB people. Cisgender researchers should also consider reflecting on questions such as who the research serves (TNB people or the researchers), whether the project will directly benefit TNB communities, and how the study portrays TNB people (Minalga et al. 2022).

### 4.2 Advancing Research on Understudied Topics

The findings from our study resonate with past research highlighting understudied areas for TNB communities, such as the importance of longitudinal research, understanding drivers of health disparities (e.g., greater research on stressors and marginalization), and the role of the broader sociopolitical context in TNB people's health (Four Corners TNB Health Research Advisory Network 2021; LeBlanc et al. 2022; Misiolek et al. 2020; Olson‐Kennedy et al. 2016; Veale et al. 2022). Although important, only studying the hardships of TNB people can create a one‐sided narrative that may inadvertently reinforce negative stereotypes about TNB people (LeBlanc et al. 2022; Levitt et al. 2023). As Tuck has written about, damage‐centered research can result in oppression being the one and only characteristic known of a community (Tuck 2009). Although we certainly need to bear witness to these hardships, research also needs to take a fuller approach to understanding TNB people's lives. Integrating a focus on trans positivity, joy, gender euphoria, and resilience can enhance research and can even impact TNB people's understandings and expectations for themselves in positive and meaningful ways (Beischel et al. 2022; Matsuno and Israel 2018; Puckett et al. 2024; Puckett et al. 2024). When researching these areas, we also caution against romanticizing resilience (Puckett et al. 2025) or emphasizing individual responses to overcoming marginalization over the importance of structural interventions to address the hardships imposed on TNB communities (Suslovic and Lett 2024). As one of our participants said, “o*ur words create realities,”* and we encourage research to continue expanding to better represent experiences of resilience and positive life experiences, while also being sensitive to how these issues are framed.

One other understudied area that was mentioned by participants is relationships, including social support, parenting, and family relationships. Social support is multifaceted and can include support from a range of people (e.g., parents, significant others, peers, friends, and others), as well as specific types of support (e.g., instrumental support, emotional support, and so forth). Even so, most TNB research about social support has used the Multidimensional Scale of Perceived Social Support to assess social support (Dowers et al. 2020). Although a helpful scale, recent critiques have raised questions about whether this scale is psychometrically sound for use with TNB participants (Barry et al. 2025).

Given the narrow scope of past research and the limited measures available, we recommend that future research establish measures of social support that are developed in collaboration with TNB communities, which may better attend to the nuances of the sources and types of support most salient to these participants. It may be that there are modifications needed to scales to make them more inclusive of TNB people's experiences (e.g., including “chosen” families as opposed to only families of origin). Alternatively, there may be unique forms of support that are specific to TNB people that are overlooked by existing measures. For example, research has shown a variety of microaggressions that may arise within friendships for TNB people, which suggests that there may be particular actions that show support within these relationships as well (Galupo et al. 2014). Through integrating TNB people's perspectives and lived experiences into social support research, we may better attend to the types of supportive relationships most core to promoting positive outcomes, learn nuanced ways that support is demonstrated, and discover better pathways to promoting the supportive relationships that matter most to community members.

### 4.3 Diverse Experiences Within TNB Communities

Participants also emphasized the importance of attending to diversity, including through advancing research on a broader range of TNB people's experiences of gender, including more nuanced understandings of paths to gender affirmation, and attending to intersectionality. In terms of gender, there are many subgroups within TNB communities, but some studies collapse all groups into a broad TNB category. For example, Puckett et al. (2021) conducted a systematic review of sexuality measurement in TNB‐focused research, and 15.6% of the studies reviewed simply described their samples as “gender minorities” or as TNB. When gender subgroups are not even named, let alone discussed in some level of detail, this neglects differences that can exist and does not provide enough information to gauge the generalizability of the samples.

Many studies also have primarily White samples, resulting in continued underrepresentation of People of Color in research (e.g., Pattar et al. 2024). When samples are mostly White, it can result in generalizing findings from White TNB people to all TNB people, which may ignore how other aspects of identity intertwine with gender to influence one's experiences (Shultz and Matsuno 2025). Through integrating an intersectional lens, researchers can explore how interlocking systems of privilege and oppression influence TNB people's experiences (Grzanka et al. 2020). One framework that researchers may use is that of Intersectionality Research for Transgender Health Justice (Wesp et al. 2019). This framework details how multiple structures of domination (e.g., cisgenderism, colonialism) interact with institutional systems (e.g., housing, criminal‐legal systems) and socio‐structural processes (e.g., racializing, criminalizing) to produce health inequities.

### 4.4 Inclusive Design and Methodologies That Better Reflect TNB People's Experiences

Beyond specific topics, participants called for an overhaul of research practices. They felt that researchers needed better awareness about best practices for working with TNB communities, more knowledge about the range of TNB identities, and more awareness about how to approach practical elements, like survey question design, with more inclusivity. There are a variety of resources available to help researchers improve their work with TNB communities and we encourage others to continue building on these recommendations in partnership with TNB communities (Adams et al. 2017; Herman et al. 2024; Puckett et al. 2018; Travers et al. 2013; Veldhuis et al. 2024; Vincent 2018). To address concerns about measures that are inclusive, it might also be beneficial to use more recent measures of study variables that are more aligned with TNB people's experiences. For example, Matsuno et al. (2024) developed a scale that focuses on nonbinary people's experiences of distal and proximal minority stressors. Other TNB‐specific measures include the Trans and Nonbinary Coping Measure (Lindley and Budge 2022) and the Multidimensional Transgender and Nonbinary Resilience Scale (Puckett et al. 2025). These measures were developed with a grounding in TNB people's experiences rather than being adaptations of measures that originated for cisgender people, which results in better representation of the lives of TNB people.

### 4.5 Moving Beyond Knowledge Generation to Tangible Impacts

One important area to attend to in terms of research practices is dissemination and making research actionable. Participants voiced wanting research to address their needs more directly rather than just documenting them. Researchers can make sure to disseminate their findings back out to TNB communities in accessible ways, send participants resources that may be helpful to them, and even use their findings to engage in advocacy efforts (Adams et al. 2017; Katz‐Wise et al. 2019; Singh et al. 2013). There can be understandable mistrust of researchers and pursuing intentional ways to ensure research benefits those who participate, and their communities, can foster stronger alliances and better center TNB people's needs over those of the researcher (Owen‐Smith et al. 2016; Singh et al. 2013). Aligned with the values of community psychology, our reach needs to extend beyond knowledge generation to making a tangible difference, driven by the communities we work with.

Likewise, others have called for greater research on intervention development (Mustanski and Macapagal 2023). There has been significant growth in the amount of TNB‐focused research (Sweileh 2018, 2022), but this has not been matched with reductions in the health disparities experienced by TNB people. Research on multilevel interventions and implementation and translational research can work towards promoting health equity and more directly impacting the lives of TNB people (Mustanski and Macapagal 2023). Through these efforts, research can move beyond knowledge production to creating practical methods and strategies for health promotion.

### 4.6 Limitations

Although this study provides insight into ways to advance TNB‐focused research, it is not without limitations. This analysis was a secondary focus of the main study, and thus, there was limited context and depth of discussion in some focus groups due to time constraints. The majority of the researchers were White, which may have affected the comfort level and vulnerability of People of Color in the study. In addition, our team did not include any researchers along the trans feminine spectrum, and thus, these perspectives may not be as salient in our reflexive analysis and may have influenced participant experiences as well. There was also an overrepresentation of low‐income and urban or suburban backgrounds in the study sample. Likewise, although our sample was majority People of Color, about 46% of participants were White. It is possible that unique areas of research that consider simultaneously one's gender and race were not as detailed in our study due to the sample demographics. For instance, past research has demonstrated unique forms of resilience at the intersection of being a racial minority and TNB (e.g., Puckett et al. 2024). Future research should seek more diverse samples, including more participants in rural areas who may have less access to contribute to research and those with a range of racial identities. Finally, the participants offered their recommendations for future research based on their own lived experiences, what they found salient or important in their lives, and, for some, based on their past experiences with research studies. Not all participants had been involved in research in the past and, for the most part, participants were also not researchers on these topics. Their reflections are valuable as they provide community‐driven insights into important topic areas, but they may not reflect an understanding of the current state of the literature or what is necessarily missing from TNB‐focused research. Instead, we have contextualized their recommendations in light of the current literature and provided recommendations based on that integration.

## 5 Conclusions

TNB community members bring important wisdom from their lived experiences that can advance and strengthen research. Recommendations from the current study include for that researchers to embrace the complexity and diversity of TNB people's lives, identities, and experiences, and to expand research areas to encompass the full lives of TNB people, with a particular focus on the positives. Also, researchers should provide tangible support to TNB people to not only incentivize but also enable TNB research involvement. Similarly, researchers should make research accessible to the TNB community and include TNB input and leadership in research that affects TNB people. In sum, it is essential for researchers to listen to TNB community members about critical areas to study in TNB research, affirming approaches to this work, and practical applications.

## Author Contributions

Jae A. Puckett was responsible for the conception and design of the study. Kye Campbell‐Fox and Arden Henderson conducted the literature review. All authors facilitated focus groups and contributed to the focus group transcription. Arden Henderson, Jae A. Puckett, and Kye Campbell‐Fox analyzed the data. Kye Campbell‐Fox, Jae A. Puckett, and Arden Henderson wrote the manuscript with contributions from all of the other authors.

## Ethics Statement

Ethical approval for this study was obtained from the Michigan State University IRB. All participants provided informed consent prior to participating in this study. No personally identifiable data is used in this paper.

## Conflicts of Interest

The authors declare no conflicts of interest.

## Data Availability

The data from this study are not publicly available.
